# Two Refractory Immune Thrombocytopenia Case Reports Showing Responsiveness to Fostamatinib

**DOI:** 10.1155/2023/9953245

**Published:** 2023-06-07

**Authors:** Vanessa Innao, Rosalba Donatella Calogero, Fabrizio Lo Presti, Ugo Consoli

**Affiliations:** U.O.C di Ematologia, Azienda Ospedaliera di Rilievo Nazionale e di Alta Specializzazione, ARNAS, Garibaldi di Catania, Catania, Italy

## Abstract

Immune thrombocytopenia (ITP) is immune-mediated platelet loss due to increased destruction and insufficient production. Treatment guidelines provide for first-line steroid-based therapies followed by thrombopoietin receptor agonists (TPO-RAs) and fostamatinib for chronic ITP. Fostamatinib demonstrated efficacy in phase 3 FIT trials (FIT1 and FIT2) mainly in second-line therapy resulting in the maintenance of stable platelet values. Here, we describe two patients with extremely heterogeneous characteristics that responded to fostamatinib after two and nine previous treatments. Responses were complete with stable platelet counts ≥50,000/*μ*L and without any grade ≥3 adverse reactions. As in the FIT clinical trials, we confirm better responses to fostamatinib when used in the second or third line. However, its use should not be excluded in patients with longer and more complicated drug histories. Given the different mechanism of action of fostamatinib compared to TPO-RAs, it would be interesting to identify predictive factors of responsiveness applicable to all patients.

## 1. Introduction

Immune thrombocytopenia (ITP) is a common autoimmune disease with an estimated incidence rate of 1.6 to 3.9 per 100,000 adult patient years and an annual prevalence of 9.5 cases per 100,000 [1]. Characterized by decreased platelet counts (PLT: <100,000/*μ*L) and increased bleeding risk, ITP is mainly due to immune-mediated platelet destruction. Differential diagnosis is important because ITP is confirmed when all other causes have been ruled out. It can arise at any age, but incidence increases with age and has a slight female preponderance.

International guidelines agree on the goal of treatment (reduced bleeding risk) and the minimum platelet values to start therapy (<20,000–30,000/*μ*L), but not on therapeutic algorithms. First-line therapies include steroids+/-intravenous immunoglobulin (IVIG), followed by thrombopoietin receptor agonists (TPO-RAs), splenectomy, rituximab, or immunosuppressants for chronic forms. Recent updates recommend the spleen tyrosine kinase (SYK) inhibitor, fostamatinib, and TPO-RAs (eltrombopag, romiplostim, and avatrombopag) in patients with chronic refractory ITP [2].

The efficacy of fostamatinib was demonstrated in phase 2 (NCT00706342) and phase 3 studies (FIT1 (NCT02076399) and FIT2 (NCT02077192)) in which just under 50% of patients achieved a stable response as defined for these trials (PLT ≥50,000/*μ*L for at least four of the six clinic visits (every two weeks) during weeks 14–24 of the study) [3–5]. Fostamatinib's novel mechanism of action is inhibition of SYK which prevents phagocytosis and destruction of platelets by macrophages and neutrophils.

## 2. Case Reports/Case Presentations

**Figure 1:**
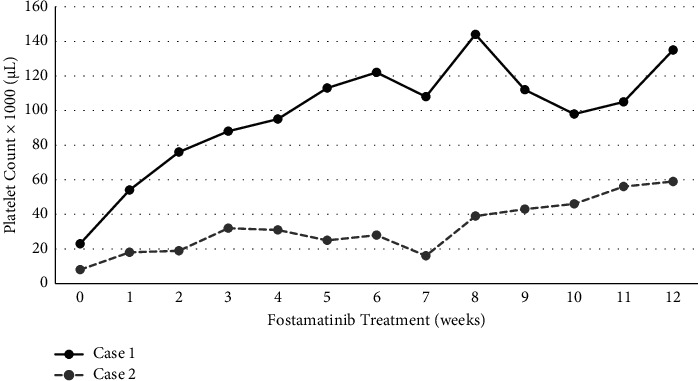
Platelet response over the first 12 weeks of fostamatinib therapy. Patient 1 (case 1) received fostamatinib as third-line therapy, obtained better quality platelet responses, and continued the drug at the initial dose of 100 mg bid. Patient 2 (case 2) was heavily pretreated, but maintains a platelet count >50,000/*μ*L with the maximum permissible dose of fostamatinib (150 mg bid).

We report the cases of two patients with chronic ITP and treated with fostamatinib in the Hematology Division of the Garibaldi Hospital in Catania.  Table 1 highlights patient characteristics, while Figure 1 shows platelet responses during the first 12 weeks of fostamatinib treatment. Compliance to treatment was assessed by the timing of prescription refills based on the dose prescribed and amount of medication dispensed. The consensus of an international working group defined CR = PLT ≥ 100,000/*μ*L) [6]. This standard will be used to evaluate responses in these cases. Adverse events were monitored by physician assessment, patient queries, and laboratory tests at clinic visits.

### 2.1. Case 1

A 54-year-old Caucasian/white male came to our clinic in January 2022. He was originally found to have moderate thrombocytopenia in 2011 (PLT ∼ 75,000/*μ*L) and was followed at another hematologic center until December 2021. Other diseases responsible for thrombocytopenia were excluded. Medical history includes hypertension well controlled with an angiotensin II inhibitor and ascending aorta ectasia (maximum diameter 40 mm).

In 2016, his platelet count was <30,000/*μ*L. A bone marrow biopsy was unremarkable. Antinuclear antibodies (ANA) were positive (1 : 80), as were antinuclear ribonucleoprotein antibodies (ENA-RNP) and anti-DNA antibodies (the latter not always confirmed). At that time, he started steroid therapy with prednisone (PDN) 50 mg/day, which he continued for ∼4-5 months with a good hematological response (defined as PLT ≥30,000/*μ*L [6] to CR: PLT max ∼ 100,000/*μ*L), but treatment was complicated by hypertension and headache. Due to a loss of response after steroid dosage reduction, at the end of 2016 he started second-line treatment with eltrombopag at an initial dose of 25 mg/day. This low initial dose was due to platelet count of 45,000/*μ*L at the start of therapy. The dose was progressively increased to 50–75 mg/day, and the patient obtained a durable response.

In September 2021, the patient was referred to a rheumatologist regarding the appearance of Raynaud's phenomenon. They suspected scleroderma, but this was excluded. In January 2022, the rheumatologist started the patient on PDN 50 mg/day for 10 days, followed by 37.5 mg/day for 10 days and then PDN 6.75 mg/day from January 20, 2022.

For concomitant worsening of ITP, the patient came to our attention on January 25, 2022. Complete blood cell counts (CBC) showed platelets at 23,000/*μ*L without additional dyscrasias. Looking at the patient's laboratory values from the previous months, his platelets were becoming progressively lower from 70,000–50,000/*μ*L to 30,000/*μ*L between October and December 2021, despite continuing eltrombopag at the maximum dosage of 75 mg/day. This was compatible with a loss of response to eltrombopag after 6 years of continuous treatment. Other laboratory tests showed no changes in hemocoagulation parameters or renal function. The patient denied noteworthy symptoms, referring exclusively to rare posttraumatic bruising. On physical examination, there were no superficial clinically relevant adenopathies noted and no hepatosplenomegaly. The severity of the hemorrhagic syndrome was quantified based on the SMOG parameters developed by the International Working Group on ITP [7]. For this patient, the SMOG value was 0.0.0.

After discussing the available therapeutic alternatives (romiplostim and fostamatinib) with the patient, he preferred the oral formulation. Informed of potential side effects and after acquisition of written informed consent, therapy with fostamatinib was started on February 2, 2022, at 100 mg twice a day for 4 weeks. Platelet count was evaluated weekly, showed a good response after two weeks (PLT 76,000/*μ*L), and improved at one month (PLT 95,000/*μ*L). At the end of the third month, there were no adverse events, blood pressure was controlled, and a CR was confirmed (PLT 135,000/*μ*L). To date, after a year of continuous treatment with fostamatinib at 100 mg twice a day, the patient maintains a CR with platelets always >100,000/*μ*L.

### 2.2. Case 2

An 80-year-old Caucasian/white female was diagnosed with acute ITP in March 2016, with isolated severe thrombocytopenia (PLT 16,000/*μ*L), and spontaneous ecchymosis spread throughout the soma (SMOG 2.0.0.). For medical history, she reported a hemithyroidectomy in 2000 due to multinodular goiter followed by hormonal replacement therapy (levothyroxine 75 mcg daily), hypertension since 2014 controlled with beta blockers, hypercholesterolemia since 2018 treated with statin therapy, renal lithiasis, and gallstones since 2016, and Parkinson's disease treated with dopaminergic agents for about three years. On admission, laboratory tests showed hepatorenal function, inflammatory indexes, and coagulative parameters within normal limits. Viral serological tests showed previous EBV and CMV infections, negative hepatitis B and C tests, and negative HIV tests. Autoimmunity testing revealed positive ANA (1 : 80) and negative antiplatelet antibodies.

In the emergency department, she was transfused with 2 units of platelets and started on high-dose steroids (initially methylprednisolone 1 mg/kg/day for four weeks, and then dexamethasone 40 mg/day for four days). Dexamethasone was chosen due to the faster response and fewer side effects compared to prednisone [6, 8]. The bone marrow biopsy results were normal. The abdominal ultrasound examination showed moderate liver steatosis. Her chest X-ray was compatible with chronic bronchopathy.

The patient came to our clinic one month after ITP diagnosis. She was continuing steroid treatment (dexamethasone, 10 mg/day). On admission, her platelet count was 23,000/*μ*L, revealing no response to dexamethasone. Treatment was initiated with intravenous immunoglobulins (IVIG) 1 g/kg/day for two consecutive days. A partial response (PLT 45,000/*μ*L) was seen on day two, so two additional IVIG cycles were given every 21 days. However, the IVIG response quickly faded after each cycle.

Therefore, at 6 months after diagnosis (October 2016), we started her on a TPO-RA, romiplostim 4 *μ*g/kg/week. After an initial response (maximum PLT 114,000/*μ*L at one month), her platelet counts gradually decreased despite maximal dosing (PLT <30,000/*μ*L at 10 *μ*g/kg/week). We decided to change the patient to second TPO-RA, eltrombopag, which was initiated on December 2016 (initial PLT 9,000/*μ*L). After one month of eltrombopag treatment, the patient's refractoriness was confirmed with PLT <10,000/*μ*L at the maximum dosage of 75 mg daily.

In January 2017, the patient underwent a splenectomy. The patient proved refractory to this procedure (PLT 3,000/*μ*L), and eltrombopag was restarted with no improvement. In sequence, IVIG cycles and weekly rituximab were given for four doses, followed by high-dose steroids, danazole, and then continuing with bimonthly cycles of IVIG + prednisone or dexamethasone until March 2021. In the meantime, in April 2018, we performed a bone marrow revaluation confirming the diagnosis.

At our center a double-blind trial with the monoclonal antibody anti-CD38, TAK-079, was ongoing, and the patient was considered eligible. The patient was screened and enrolled. On May 18, 2021, she started TAK-079 treatment (PLT 6,000/*μ*L). Weekly intravenous infusions were given for 8 weeks. The patient obtained a minimal response (maximum PLT 16,000/*μ*L after four infusions). During the subsequent off-treatment observational period of 16 weeks, the patient remained severely thrombocytopenic (PLT <10,000/*μ*L).

After the approval of fostamatinib for patients with refractory chronic ITP, we started treatment at 100 mg twice a day for 4 weeks (initial PLT 8,000/*μ*L). Platelet count was evaluated weekly, showing a mild response at one month (PLT 32,000/*μ*L). Fostamatinib was increased to 150 mg twice a day giving a stable response after 6 weeks at the full dose (PLT 56,000/*μ*L). Stable response is defined here as in the fostamatinib FIT1 and FIT2 trials (PLT >50,000/*μ*L) [4, 5]. The International Working Group defined a complete response as PLT >100,000/*μ*L [7]. At three months, there were no adverse events and blood pressure was stable and normalized. A stable response was confirmed (PLT 59,000/*μ*L) and no bleeding has been reported. After six months of treatment, the patient's complete response was confirmed (PLT >200,000) and the dosage of fostamatinib was reduced to 100 mg twice a day. To date, after a year of continuous treatment with fostamatinib, the dosage was reduced at 100 mg per day due to an excessive increase in platelets (PLT >400,000/*μ*L).

## 3. Discussion/Conclusion

These two cases illustrate how seemingly unfavorable characteristics (long ITP history, numerous prior therapies, and refractoriness) do not necessarily impact therapeutic outcomes. Moreover, fostamatinib can stabilize platelet counts without vigorous platelet responses, reduce the risk of bleeding, and reduce the need for rescue therapy [4, 5]. Even in patients without a partial or complete response, fostamatinib provided independence from rescue therapies and improvement in SMOG scores [7]. In addition, fostamatinib has been found to be well tolerated. The most common adverse events associated with fostamatinib treatment (diarrhea, nausea, hypertension, and transaminase elevation) have been mild to moderate in intensity [9, 10].

Current American Society of Hematology guidelines briefly mention fostamatinib as it had only recently been approved when the guidelines were issued and had been studied primarily in a third-line setting [11]. Recommended first-line therapy for adults was corticosteroids ± immunoglobulins followed by second-line therapy with rituximab, TPO-RA, or splenectomy based on assessment of the patient's physical and social conditions and preferences.

Despite a modest platelet response, early use of fostamatinib as a second- or third-line agent can be justified in patients with underlying prothrombotic conditions or with previous thrombosis for whom TPO-RAs may not be safe and anti-CD antibodies (rituximab) or splenectomy are inadvisable due to the risk of immunosuppression or surgical complications. However, the lack of randomized, comparative clinical trials does not allow effective evaluation of the best treatment sequence in ITP patients. Extrapolation from case reports and comparisons between studies do not provide the highest quality data to drive therapeutic decisions.

In addition, the best responses obtained from the second line onwards should stimulate clinicians to stratify patients according to risk factors adapting therapy to their clinical conditions. If thrombotic events are a concern, a procoagulant profile may be useful. Increases in prothrombin fragments 1 and 2, plasminogen activator inhibitor-1 (PAI-1), and D-dimer could increase thrombotic risk [12]. This stratification before starting second-line therapies could favor fostamatinib over TPO-RAs for patients with high thrombotic risk given fostmatinib's reported protection from thromboembolism [13]. Decision-making may be complicated in patients with previous thrombotic events on anticoagulant therapy and for whom platelet counts >50–80,000/*μ*L are recommended [14]. In these cases, it may be useful to discuss with the cardiologist the possibility of using fostamatinib with an anticoagulant with low hemorrhagic risk (e.g., fondaparinux or apixaban) [15].

## Figures and Tables

**Table 1:** Patient characteristics.

	Age, sex, and race	Year of ITP diagnosis	Concomitant conditions	Concomitant medications	Time from diagnosis to fostamatinib Tx (years)	No. of previous ITP treatments	Type of previous ITP treatments^a^	Response to previous therapies	Baseline platelets, before fostamatinib (microliter)
Case 1	54, M, Caucasian	2011	Hypertension Ascending aorta ectasia	Sartan derivative	11	2	Steroids Eltrombopag	PR Loss of CR	23,000

Case 2	80, F, Caucasian	2016	Multinodular goiter Hypertension Hypercholesterolemia Renal lithiasis and gallstone Parkinson's disease	Levothyroxine Β-adrenergic blocking agents Statins Dopaminergic agents	6	9	Steroids (PDN and Dexa) IVIG Romiplostim Eltrombopag Splenectomy Rituximab Danazole (+Dexa) IVIG + PDN Anti-CD38 (TAK-079)	PR PR Brief CR Refractoriness Refractoriness Refractoriness Refractoriness Refractoriness Refractoriness	6,000

M = male, F = female, PDN = prednisone, Dexa = dexamethasone, IVIG = intravenous immunoglobulins, PR = partial response, and CR = complete response. ^a^Treatments are reported in the order prescribed.

## Data Availability

All data generated or analyzed during this study are available from the corresponding author upon reasonable request.

## Supplementary Materials

Supplementary Materials The CARE Checklist of case report guidelines has been submitted as supplementary material to this manuscript.
