# Supplementary material for: Two Refractory Immune Thrombocytopenia Case Reports Showing Responsiveness to Fostamatinib
Source: Case Rep Hematol. 2023 Jun 7;2023:9953245. doi: 10.1155/2023/9953245 (PMC10266907; doi:10.1155/2023/9953245)
Supplement: Supplementary Materials — The CARE Checklist of case report guidelines has been submitted as supplementary material to this manuscript. [file 9953245.f1.pdf]

CARE Checklist of information to include when writing a case report

CARE case report guidelines

(cc) BY-NC-ND

Topic	Item	Checklist item description	Reported on Line
Title	1	The diagnosis or intervention of primary focus followed by the words "case report"	Title page
Key Words	2	2 to 5 key words that identify diagnoses or interventions in this case report, including "case report"	Title page
Abstract (no references)	3a	Introduction: What is unique about this case and what does it add to the scientific literature?	1-30
	3b	Main symptoms and/or important clinical findings	21-51, 67-81
	3c	The main diagnoses, therapeutic interventions, and outcomes	52-65, 82-109
	3d	Conclusion—What is the main "take-away" lesson(s) from this case?	111-134
Introduction	4	One or two paragraphs summarizing why this case is unique (**may include references**)	111-116
Patient Information	5a	De-identified patient specific information.	36-109
	5b	Primary concerns and symptoms of the patient.	36-40,67-76
	5c	Medical, family, and psycho-social history including relevant genetic information	36-40,67-76
	5d	Relevant past interventions with outcomes	41-58, 77-103
Clinical Findings	6	Describe significant physical examination (PE) and important clinical findings.	36-58, 67-103
Timeline	7	Historical and current information from this episode of care organized as a timeline	59-65, 104-109
Diagnostic Assessment	8a	Diagnostic testing (such as PE, laboratory testing, imaging, surveys).	36-58, 67-103
	8b	Diagnostic challenges (such as access to testing, financial, or cultural)	NA
	8c	Diagnosis (including other diagnoses considered)	36-40, 67-76
	8d	Prognosis (such as staging in oncology) where applicable	NA
Therapeutic Intervention	9a	Types of therapeutic intervention (such as pharmacologic, surgical, preventive, self-care)	43-65, 77-109
	9b	Administration of therapeutic intervention (such as dosage, strength, duration)	43-65, 77-109
	9c	Changes in therapeutic intervention (with rationale)	54-62,83-102
Follow-up and Outcomes	10a	Clinician and patient-assessed outcomes (if available)	52-65, 82-109
	10b	Important follow-up diagnostic and other test results	52-65, 82-109
	10c	Intervention adherence and tolerability (How was this assessed?)	34-37
	10d	Adverse and unanticipated events	36-37
Discussion	11a	A scientific discussion of the strengths AND limitations associated with this case report	117-134
	11b	Discussion of the relevant medical literature **with references**.	111-134
	11c	The scientific rationale for any conclusions (including assessment of possible causes)	111-134
	11d	The primary "take-away" lessons of this case report (without references) in a one paragraph conclusion	124-134
Patient Perspective	12	The patient should share their perspective in one to two paragraphs on the treatment(s) they received	NA
Informed Consent	13	Did the patient give informed consent? Please provide if requested	Yes ☑ No ☐
